# Phages Affect Gene Expression and Fitness in E. coli

**DOI:** 10.1371/journal.pbio.0030258

**Published:** 2005-06-21

Life is hard for bacteria. Not only must they constantly compete against their comrades for resources and living space, they're also subject to infection by pathogens—viruses called bacteriophages—which can affect their ability to survive and prosper. Two types of bacteriophages threaten bacteria: lytic phages and lysogenic (or temperate) phages. Acquisition of a lytic phage (for example, T2, T4, or T6) is an immediate death sentence for the bacterium; upon infection, a lytic phage subverts the bacterium's biochemical machinery to make copy after copy of itself until the bacterium bursts, or lyses, from the burden. In contrast, a temperate phage (for example, λ phage) can lie dormant for many generations before it co-opts the bacterium's machinery to reproduce, but eventually it, too, lyses the bacterial cell as it releases a host of new phages. From the perspective of the bacterium, it is better to be infected by a temperate phage than a lytic phage because infection with a lytic phage means instant death, while a temperate phage may lie dormant long enough for the bacterium to reproduce. [Fig pbio-0030258-g001]

**Figure pbio-0030258-g001:**
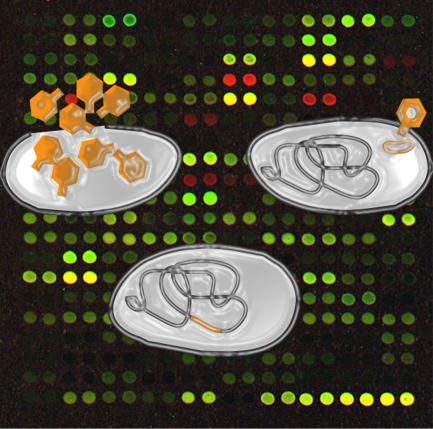
The bacterial virus λ integrates into the E. coli genome, where it shuts down the cell's ability to grow on poor carbon sources

Temperate phages achieve dormancy by producing a phage gene product (in the case of λ phage, called cI) that represses the production of other phage genes; phage reproduction ceases as long as this repressor is produced. Once infected by a temperate phage, bacteria are protected from secondary infections by various other phages, because the temperate phage prevents the others from becoming established in the cell. But might temperate phage infection confer other advantages on bacterial survival?

Edward Cox's group at Princeton University examined this question by looking for evidence that temperate phage infection triggers changes in bacterial behavior. Working with λ phages, the authors studied how phage infection affects the regulation of genes that might impact the bacterium's survival by comparing the constellation of genes expressed in uninfected E. coli bacteria to those in E. coli carrying a dormant λ phage. They found that λ phage caused reduced expression of the bacterial gene *pckA*, which codes for an enzyme that helps bacteria grow on carbon sources (fuels) other than glucose; without functioning *pckA*, bacteria grow normally in an environment containing glucose, but grow only slowly in an environment containing alternative carbon sources such as succinate. E. coli carrying λ phage fail to make the *pckA* gene product because the *pckA* gene is turned off by the virally encoded repressor cI. Interestingly, the researchers found evidence that the repressors made by other temperate phages may also be able to turn off *pckA* expression, and that the *pckA* genes of other bacteria related to E. coli might also be regulated by temperate phage repressors.

The fact that this relationship between temperate phage repressors and regulation of the *pckA* gene is so well conserved argues that the ability to turn off this gene might be positively selected; therefore, *pckA* repression must confer some sort of survival benefit to the bacterium. It's not clear what this benefit might be, but one explanation is that slowing bacterial growth in glucose-poor environments might help the bacterium elude detection by the immune system of any animal it invades, increasing its chances of survival. Alternatively, slower bacterial growth might slow down the onset of viral reproduction and eventual lysis. Regardless, it is clear that there is a strong relationship between the temperate phages and the bacteria they colonize. These results have significant implications for the evolution of fitness in these bacterial populations.

